# Frailty in emergency surgery: expanding the role of biomarkers

**DOI:** 10.1308/rcsann.2026.0051

**Published:** 2025-11-26

**Authors:** HJ Ng, N Rattray, T Quasim, S Moug

**Affiliations:** ^1^ NHS Greater Glasgow and Clyde, UK; ^2^ University of Glasgow, UK; ^3^ University of Strathclyde, UK

**Key points**

• Frailty is a major determinant of outcomes in emergency laparotomy but is incompletely captured by current clinical assessment tools.
• It should be viewed as a dynamic state that interacts with surgical stress and influences recovery trajectories.
• Biomarkers from multiomics platforms offer objective insight into the biological mechanisms underlying frailty although current evidence is based largely on non-surgical populations.
• Integration of biomarkers with clinical assessment may enable personalised perioperative care and targeted interventions to improve outcomes.

Emergency laparotomy (EmLap) represents one of the most challenging procedures in acute surgical care. Despite improvements in perioperative management and national quality improvement initiatives, outcomes remain highly variable. Mortality rates following EmLap continue to be substantial, reflecting both the severity of underlying pathology and the vulnerability of patients undergoing surgery. A major determinant of these outcomes is the presence of frailty. Frailty is widely recognised as a multidimensional clinical syndrome characterised by reduced physiological reserve and diminished capacity to respond to stressors.^1^ In surgical populations, frailty has been associated with increased postoperative complications, prolonged hospitalisation, delayed recovery and higher mortality.^2^

In emergency surgical settings, the interaction between frailty and physiological stress is particularly pronounced. Patients presenting for EmLap often have acute pathologies such as bowel perforation, obstruction or ischaemia.^3^ These conditions impose considerable physiological stress even before surgical intervention, further compounding the vulnerability associated with frailty.

Although clinical frailty assessments are increasingly incorporated into perioperative risk evaluation, they do not fully explain the biological mechanisms underlying frailty. Consequently, there is rising interest in identifying biomarkers that reflect the underlying physiological processes associated with frailty. Such biomarkers may provide objective insights into patient resilience, improve risk stratification and support the development of personalised perioperative strategies. In addition, biomarker-informed approaches may facilitate targeted functional and pharmacological interventions aimed at enhancing physiological resilience, improving functional recovery, and optimising both surgical and patient-centred outcomes in frail surgical patients.

## Clinical assessment of frailty in surgical practice

Frailty assessment has gained increasing prominence in perioperative care over the past decade. Several tools have been developed to identify frailty in clinical settings, including the frailty phenotype, deficit accumulation models and multidimensional clinical scales. Among these, the clinical frailty scale (CFS) has become one of the most widely used instruments in acute clinical environments. Originally developed by Rockwood *et al*,^4^ the CFS evaluates functional status across a spectrum ranging from very fit to severely frail, incorporating mobility, functional independence, comorbidities and cognitive function.

In emergency surgical pathways, frailty assessment serves several important purposes. Preoperatively, it supports risk stratification and facilitates shared decision making between clinicians, patients and families. Postoperatively, repeated frailty assessment can help identify patients experiencing early functional decline and guide targeted rehabilitation strategies. However, the predictive capacity of individual frailty markers remains variable. Single-domain indicators such as sarcopenia, cognitive impairment or nutritional status may reflect important components of frailty but often fail to capture the complexity of frailty as a whole. Moreover, the intense physiological stress that accompanies EmLap may overshadow subtle differences in baseline vulnerability. These limitations highlight the need for complementary approaches capable of capturing the underlying biological processes associated with frailty.

## Frailty as a dynamic physiological state

Frailty has traditionally been conceptualised as a fixed preoperative characteristic. Nevertheless, growing evidence suggests that frailty represents a dynamic physiological state influenced by ageing, chronic disease, environmental factors and acute stressors.^5^ EmLap represents a major physiological stress event. The surgical intervention, systemic inflammatory responses and the metabolic demands of acute illness place significant strain on already limited physiological reserves.

The dynamic model proposed by Clegg *et al* conceptualises frailty as progressive decline in physiological reserve over time, punctuated by stress events that may accelerate deterioration.^5^ Within this framework, EmLap can act as a tipping point that precipitates further decline in vulnerable individuals.

Longitudinal observations in surgical populations support this concept, demonstrating that frailty status frequently worsens following major emergency surgery. These findings reinforce the concept that frailty is not merely a pre-existing condition but rather a dynamic state that evolves throughout the perioperative period.

## Biomarkers of frailty in surgical patients

The search for objective biomarkers of frailty has become an important area of research in perioperative medicine. Biomarkers offer measurable insight into biological processes underlying vulnerability and may complement traditional clinical frailty assessments. However, it is important to recognise that most biomarker research in frailty has been conducted in community-dwelling older adults rather than in surgical populations. While these studies have greatly advanced our understanding of the biological mechanisms underlying frailty, their applicability to acute surgical settings remains uncertain. Patients undergoing EmLap are exposed to considerable physiological stress from both the underlying pathology and the operative intervention itself, which may alter biomarker profiles and influence frailty trajectories in ways that differ from community-based populations.

Several biological pathways have been implicated in frailty, including chronic inflammation, immune dysfunction, mitochondrial impairment, endocrine dysregulation and muscle degradation.^6^^–^^9^ Biomarkers reflecting these processes may therefore prove to be valuable indicators of reduced physiological resilience.

Inflammatory markers represent some of the most widely studied biomarkers associated with frailty. Elevated levels of cytokines such as interleukin 6 and tumour necrosis factor have been linked to frailty and poor surgical outcomes.^6^ Chronic low-grade inflammation (often referred to as inflamm-ageing) is believed to contribute to frailty through its effects on muscle wasting, immune dysfunction and metabolic instability.^7^

Markers of muscle metabolism have also received increasing attention. Sarcopenia, characterised by progressive loss of skeletal muscle mass and function, represents a major contributor to frailty.^8^ Consequently, biomarkers associated with muscle breakdown, protein metabolism and energy regulation may provide insight into frailty-related vulnerability. Other potential biomarkers include indicators of oxidative stress, mitochondrial function and cellular repair mechanisms.^10^ These biological processes are closely linked to ageing and may contribute to reduced physiological resilience during surgical stress.

Recent advances in biological technologies such as metabolomics, proteomics, transcriptomics and other ‘omics’ approaches have expanded the scope of frailty biomarker research. These emerging platforms have enabled comprehensive profiling of biological systems and provided deeper understanding into the molecular mechanisms underlying frailty. Metabolomic technologies in particular have gained attention for their ability to capture dynamic changes in metabolic pathways associated with ageing, inflammation and physiological stress. These approaches offer an opportunity to identify novel biomarker signatures that reflect the complex biological processes contributing to frailty and surgical vulnerability.

Biomarker research also highlights the biological heterogeneity present in surgical populations. Patients undergoing EmLap present with diverse underlying pathologies, each with distinct inflammatory and metabolic responses. Understanding these biological differences may enable more personalised approaches to perioperative care.

## Future research directions

Although substantial progress has been made in understanding frailty in surgical populations, several important research priorities remain. First, future work should focus on integrating biomarker data with established clinical frailty assessment tools to develop more comprehensive perioperative risk stratification models. Given that frailty reflects complex interactions between inflammatory, metabolic, endocrine and musculoskeletal systems, it is unlikely that a single biomarker will capture this complexity adequately. Multimodal approaches incorporating panels of biomarkers alongside clinical assessment may therefore offer greater predictive accuracy.

Second, dedicated research in surgical populations is needed. Much of the current biomarker evidence derives from community-dwelling cohorts and the relevance of these findings in acute surgical settings remains uncertain. Patients undergoing emergency surgery are a heterogeneous population that experience profound physiological stress, which may alter biomarker profiles and influence recovery trajectories.

With the advances in technologies, new opportunities are available for expanding biomarker discovery in this field. Multiomics platforms may help identify molecular signatures associated with physiological resilience and postoperative recovery, improving understanding of the biological basis of frailty. These omics platforms may also support the development of targeted therapeutic strategies aimed at improving physiological resilience in frail patients. Increasing attention has been directed towards pharmacological interventions that modulate inflammatory pathways, mitochondrial function and metabolic homeostasis. Potential strategies include anti-inflammatory therapies, metabolic modulators, and nutritional interventions designed to support muscle metabolism and cellular energetics. Although these approaches remain largely investigational, biomarker-guided therapeutic strategies represent an important step towards precision perioperative medicine.

Another important consideration is the heterogeneity of emergency surgical populations. EmLap encompasses a wide range of pathologies, each associated with distinct inflammatory responses. For this reason, future studies should consider stratifying patients according to both frailty status and underlying pathology.

Finally, large multicentre studies will be required to validate candidate biomarkers and determine their predictive value for surgical outcomes. Integration of biomarker research with national surgical registries may enable large-scale analysis of perioperative trajectories and support the development of biologically informed care pathways for frail surgical patients.

## Conclusions

Frailty has emerged as a critical determinant of outcomes in emergency surgery. Growing evidence suggests that frailty should be understood not merely as a clinical descriptor but as a biologically grounded state characterised by impaired physiological reserve and vulnerability to stress.

Clinical frailty assessment tools provide valuable insight into patient vulnerability but do not fully capture the biological mechanisms driving adverse outcomes. Biomarker research offers an important opportunity to bridge this gap by identifying measurable indicators of physiological resilience. However, much of the current biomarker evidence derives from community-dwelling populations and further research is required to establish its relevance in surgical patients.

Advances in biological technologies, including metabolomic and other multiomics platforms, provide new opportunities to explore the molecular mechanisms underlying frailty and surgical vulnerability. Integrating these approaches with established frailty assessment tools may improve risk stratification, enable personalised perioperative care and support the development of targeted interventions aimed at improving recovery in vulnerable patients.
